# Genomic characterization of endemic diarrheagenic *Escherichia coli* and *Escherichia albertii* from infants with diarrhea in Vietnam

**DOI:** 10.1371/journal.pntd.0011259

**Published:** 2023-04-04

**Authors:** Atsushi Iguchi, Taichiro Takemura, Yoshitoshi Ogura, Thi Thu Huong Nguyen, Taisei Kikuchi, Miki Okuno, Asako Tokizawa, Hanako Iwashita, Hong Quynh Anh Pham, Thi Hang Doan, Na Ly Tran, Thi Luong Tran, Thi Hang Nguyen, Thi Hien Tran, Tuyet Ngoc Linh Pham, Trung Duc Dao, Thi My Hanh Vu, Thi Nga Nguyen, Hieu Vu, Van Trang Nguyen, Thi Thu Huong Vu, Thanh Huong Le, Tuan Anh Lai, Tuan Cuong Ngo, Futoshi Hasebe, Dong Tu Nguyen, Tetsu Yamashiro

**Affiliations:** 1 Department of Animal and Grassland Sciences, Faculty of Agriculture, University of Miyazaki, Miyazaki, Japan; 2 Center for Animal Disease Control, University of Miyazaki, Miyazaki, Japan; 3 Vietnam Research Station, Institute of Tropical Medicine, Nagasaki University, Nagasaki, Japan; 4 Division of Microbiology, Department of Infectious Medicine, Kurume University School of Medicine, Fukuoka, Japan; 5 Thai Nguyen university of Agriculture and Forestry, Thai Nguyen, Vietnam; 6 Department of Infectious Disease, Faculty of Medicine, University of Miyazaki, Miyazaki, Japan; 7 Department of Integrated Biosciences, Graduate School of Frontier Sciences, The University of Tokyo, Chiba, Japan; 8 Department of Hygiene and Public Health, Tokyo Women’s Medical University, Tokyo, Japan; 9 Bacteriology Department, National Institute of Hygiene and Epidemiology, Hanoi, Vietnam; 10 National Institute for Control of Vaccines and Biologicals, Ministry of health, Hanoi, Vietnam; 11 Center for Disease Control, Nam Dinh, Vietnam; 12 Department of Bacteriology, Graduate School of Medicine, University of the Ryukyus, Okinawa, Japan; Institut Pasteur, FRANCE

## Abstract

**Background:**

Diarrheagenic *Escherichia coli* (DEC) is a group of bacterial pathogens that causes life-threatening diarrhea in children in developing countries. However, there is limited information on the characteristics of DEC isolated from patients in these countries. A detailed genomic analysis of 61 DEC-like isolates from infants with diarrhea was performed to clarify and share the characteristics of DEC prevalent in Vietnam.

**Principal findings:**

DEC was classified into 57 strains, including 33 enteroaggregative *E*. *coli* (EAEC) (54.1%), 20 enteropathogenic *E*. *coli* (EPEC) (32.8%), two enteroinvasive *E*. *coli* (EIEC) (3.3%), one enterotoxigenic *E*. *coli* (ETEC), and one ETEC/EIEC hybrid (1.6% each), and surprisingly into four *Escherichia albertii* strains (6.6%). Furthermore, several epidemic DEC clones showed an uncommon combination of pathotypes and serotypes, such as EAEC Og130:Hg27, EAEC OgGp9:Hg18, EAEC OgX13:H27, EPEC OgGp7:Hg16, and *E*. *albertii* EAOg1:HgUT. Genomic analysis also revealed the presence of various genes and mutations associated with antibiotic resistance in many isolates. Strains that demonstrate potential resistance to ciprofloxacin and ceftriaxone, drugs recommended for treating childhood diarrhea, accounted for 65.6% and 41%, respectively.

**Significance:**

Our finding indicate that the routine use of these antibiotics has selected resistant DECs, resulting in a situation where these drugs do not provide in therapeutic effects for some patients. Bridging this gap requires continuous investigations and information sharing regarding the type and distribution of endemic DEC and *E*. *albertii* and their antibiotic resistance in different countries.

## Author summary

Diarrhea is a leading cause of morbidity and mortality in children under five years of age in developing countries, and diarrheagenic *Escherichia coli* (DEC) is a group of bacterial pathogens that cause a wide variety of intestinal diseases. To control the epidemic of DECs, it is necessary to understand the characteristics of prevalent strains in each region and propose appropriate treatment methods for them. This study reports detailed genomic analysis results of DEC-like strains isolated from infant diarrhea patients in Vietnam. The results suggested that many of the DECs prevalent in this country are uncommon types, and that *Escherichia albertii*, an emerging diarrheal pathogen, is the cause of diarrhea. Furthermore, comprehensive genome analysis revealed that these DECs are developing serious drug resistance. We believe that the results of this study will be the basis for improving treatment methods for patients with diarrhea and for tracking the evolution of this pathogenic group.

## Introduction

Diarrhea is a leading cause of morbidity and mortality in children under five years of age in developing countries, especially in South-Asia and Africa, where the accessibility to safe water, good nutrition, adequate sanitation, and proper healthcare is restricted [1]. Vietnam, located in South-Asia, has achieved rapid economic growth in recent years. The infrastructure, medical systems, and food production and food marketing systems that contribute to the improving public health are being developed. However, childhood diarrhea remains a public health problem in Vietnam [2].

The enteric pathogens that cause diarrhea include various viruses, bacteria, and parasites. Among them, diarrheagenic *Escherichia coli* (DEC) is a group of bacterial pathogens that causes a wide variety of intestinal diseases. DEC is generally classified into at least five pathotypes, including Shiga toxin-producing *E*. *coli* (STEC), enteropathogenic *E*. *coli* (EPEC), enterotoxigenic *E*. *coli* (ETEC), enteroaggregative *E*. *coli* (EAEC), and enteroinvasive *E*. *coli* (EIEC) based on the specific virulence markers [3]. Among the DEC pathotypes, EAEC, ETEC, and EPEC are generally associated with childhood diarrhea in developing countries [4,5].

Some studies of DEC in Vietnamese children have been conducted so far [6–8]. Nguyen et al. [6] reported that one or more marker genes of DEC were detected in 22.5% of fecal samples (132/587) collected from young children with diarrhea from 2001 to 2002. Meanwhile, Hien et al. [7] isolated DEC strains from 25.7% (64/249) of stool samples of diarrhea patients who were less than five years of age from 2001 to 2002 and classified them into pathotypes. Recently, Duong et al. [8] developed new multiplex real-time polymerase chain reactions (PCRs), which revealed that 34.7% and 41.2% of stool samples from children with and without diarrhea from 2014 to 2016, respectively, were positive for the DEC pathotypes. Thus, Vietnamese children are widely contaminated with various DEC strains possibly associated with diarrhea. However, there is little information about the characteristics of these DECs that would be useful for future epidemiological research and infection control.

Genome analysis of DEC reveals the overall picture of prevalence and the repertoires of virulence-related and antibiotic-resistance genes. It can also clarify the phylogenetic relationships between strains, and these can then be used for public health investigations. We performed a detailed genomic analysis of 61 DEC-like isolates from children with diarrhea in Northern Vietnam to clarify and share the characteristics of the DEC strains prevalent in Vietnam.

## Materials and methods

### Ethics statement

Research approval was obtained from the Ethical Committee of the Institute of Tropical Medicine Nagasaki University in Japan (approval number: 150917144) and the Institutional Review Board of the National Institute of Hygiene and Epidemiology (NIHE) in Vietnam (approval number: IRB-VN1059-19). Written informed consent was obtained from all parents of participant children.

### Isolation of DEC

Nine-hundred and ninety fecal samples were obtained from children less than five years old with diarrhea who visited the Nam Dinh Children’s Hospital in Nam Dinh province, Northern Vietnam, from 2012 to 2015. Diarrhea duration before the examination was ≤ 4 days, and the diarrhea frequency ranged from 1 to 3 times a day. Symptoms included watery diarrhea, and no patient had bloody stools. The samples were inoculated onto MacConkey agar plates and incubated for 18 to 24 hours at 35°C. Single colonies (1 to 5 colonies in each sample) grown on the plate were screened via PCR, targeting seven *E*. *coli* pathotype marker genes; *stx1* and *stx2* (encoding Shiga toxin 1 and Shiga toxin 2, respectively) for STEC [9], *eae* (encoding intimin) for EPEC [10], *elt* and *est* (encoding heat-labile enterotoxin and heat-stable enterotoxin, respectively) for ETEC [11], *aggR* (encoding a transcriptional activator of several EAEC virulence genes) for EAEC [12], and *ipaH* (encoding invasion plasmid antigen H) for EIEC [13]. When multiple strains from the same sample possessed the same marker gene(s), one positive strain was randomly selected, and stored at -80°C. The stored strains were re-cultured in LB broth for genome analysis, and the presence or absence of the corresponding genes was reconfirmed using the same PCR method.

### Genome sequencing, assembly, and annotation

Draft genomes were determined using a MiSeq sequencer (Illumina, San Diego, CA, USA). Illumina short-read libraries were prepared from 100 ng of extracted DNA using the Nextera DNA Library Prep Kit, and paired-end reads were generated using the MiSeq Reagent Kit (v3-600) and MiSeq (Illumina). Raw reads were trimmed by Platanus trim [14] with default parameters and genome assembly was performed using the Platanus_B assembler ver. 1.2.2 [14]. Annotation was carried out using Prokka ver. 1.13 with the default settings [15]. The sequence data have been deposited in NCBI under the accession number, BioProject PRJDB14289.

### Phylogenetic tree, ST typing, and phylogrouping

Core genes were identified using Roary ver. 1.2.3 [16] with the following options: -i 80 -cd 100 -s. Single nucleotide polymorphism (SNP) sites were extracted from the core gene alignment using snp-sites ver. 2.3.2 [17], and maximum likelihood (ML) phylogenomic trees were constructed using RAxML ver 8.2.10 [18] with the GTR-GAMMA model of nucleotide substitution and 500 bootstrap replicates. The ML phylogenomic trees were displayed and annotated using iTOL ver. 6 [19]. Sequence types (STs) were determined with MLST 2.0 in the Center for Genomic Epidemiology (CGE) (http://www.genomicepidemiology.org/) using assembled sequences. Phylogroup was determined using ClermonTyping ver. 20.03 [20].

### Prevalence of virulence-related and antibiotic-resistance genes

Virulence-related genes and antibiotic-resistance genes were identified from the VFDB [21] and CARD [22] databases, respectively, using SRST2 ver. 0.2.0 [23] with the default settings. Point mutations on *gyrA* and *parC* involved in drug resistance were determined with ResFinder 4.1 using assembled sequences, within the CGE.

### DNA-based serotyping

DNA-based O:H serotype (Og:Hg) was determined using the following marker gene sequences: *wzx*/*wzy* and *wzm*/*wzt* from typical *E*. *coli* O-serogroups O1 to O187 [24], a set of *Shigella* O-serogroups including *S*. *boydii* type 16/*E*. *coli* O188 [25], and *E*. *coli* OX serogroups (OX13, OX18, OX25, and OX28) [26]; Og5413 [27], OgS88 [28], and recently defined novel Og types such as OgN1, OgN8, OgN9, OgN10, OgN12, and OgN31 from STEC [29]; OgN32, OgN33, OgN34, and OgN48va from STEC [30]; OgN-RKI1, OgN-RKI3, and OgN-RKI4 from STEC [31]; OgN2, OgN4, OgN5, OgN13, OgN14, OgN15, OgN16, and OgN17 from ETEC [32]; and *fliC* and its homologs including: *flkA*, *fllA*, *flmA*, and *flnA* from typical *E*. *coli* H types H1 to H56 [33]. Several different O-serogroup strains possess highly homologous O-antigen biosynthesis gene clusters including marker genes that are difficult to distinguish by nucleotide sequences [24]. In this DNA-based serotyping, we used a previously proposed genotypes namely, OgGp1 to OgGp15 as they harbor highly homologous marker genes (≥ 97%) [24]. OgGp5, OgGp9, and OgGp7 consist of O-groups O123 and O186, O2 and O50, and O17, O44, O73, O77, and O106, respectively. A homology search was performed using BLASTN with 90% overlap and 70% identity thresholds. In addition, marker genes from *E*. *albertii* (EAOg) were also used [34] with the same overlap and threshold. In addition, it was confirmed that the paired marker genes; *wzx*/*wzy* or *wzm*/*wzt* located in the same contig (the same O-antigen biosynthesis gene cluster). If not classified into any type, it was described as Og untypeable (OgUT) or Hg untypeable (HgUT), respectively. Based on the representative sequences of 30 intimin subtypes containing α1, α2, α8, β1, β2, β3, γ1, γ2, ε1, ε2, ε3, ε4, ξ, z, z3, η, η2, θ, τ, ι1, ι2, κ, λ, μ, ν, υ, ο, π, ρ, and σ, the subtype of each *eae* was determined using BLASTN with 90% overlap and 70% identity thresholds [35].

### Statistical analysis

Fisher’s exact test for comparing between two groups was performed with EZR (ver. 1.55) and R statistical software package (ver. 4.1.3) [36]. A *P*-value < 0.05 was considered as significant for statistical analysis.

## Results

### DEC strains

DEC was isolated from 122 of the 990 samples (12.3%), including 64 *aggR*- (6.5%), 8 *ipaH*- (0.8%), 47 *eae-* (4.7%), 2 *est*- (0.2%), and 1 *elt*-positive (0.1%) strains. Neither *stx1* nor *stx2*-positive strains were detected in any of the samples. No strains harboring different marker genes were isolated from the same sample. Of the 122 strains, 8, 65, 38 and 11 were isolated in years 2012, 2013, 2014, and 2015, respectively (S1 Table). The strains stored at -80°C for a long period after isolation were re-cultured, and the virulence gene was confirmed via PCR. Unfortunately, 29 strains were not confirmed to grow in an appropriate medium, and 16 strains were not confirmed to retain the virulence genes identified at the time of isolation (S1 Table). When the genome sequences of the remaining 77 strains were determined, 16 strains did not maintain sufficient quality for genome analysis (S1 Table). Finally, draft genomes of 61 strains from 61 patients (1 to 55 months old) excluding the deficient strains were used for detailed genome analysis (S1 and S2 Tables).

### Pathotypes and phylogenetic features

**Fig 1:**
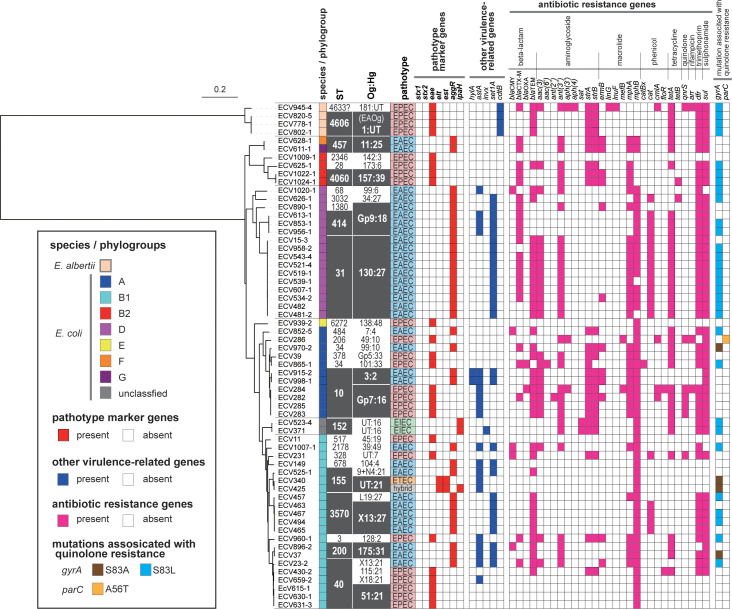
Phylogenetic tree, sequence type (ST), DNA-based serotype (Og:Hg), and prevalence of virulence-related and antimicrobial resistance genes/mutations. The ML tree was constructed based on 254,023 SNP sites in 2,197 core genes. Other virulence-related genes and antibiotic-resistance genes that were negative in all strains tested are not shown. An asterisk on ST indicates a single base mismatch or incomplete duplication on one gene compared to the reference sequence. UT: untypeable.

Genome size (total scaffold length) ranged from 4.64 to 5.96 Mb (average: 5.16 Mb), and the coverage of the obtained nucleotide sequences (total read length / total scaffold length) was 25 to 90 (S2 Table). Phylogenetic analysis revealed that 57 strains (93.4%) were classified as *E*. *coli*. While, the remaining four (6.6%) were unexpectedly identified as *E*. *albertii*, which substantially differs from the *E*. *coli* group (Fig 1). All four *E*. *albertii* strains were positive for *eae*. Three strains (ECV778-1, ECV820-5, and ECV902-1) were classified as EAOg1:HgUT and possessed *eae* subtyped as the σ (sigma) subtype. The remaining one (ECV945-4) was classified as Og181:HgUT (which is a common Og-type with *E*. *coli*) and possessed *eae* subtyped as ε3 (epsilon 3) (Fig 1 and S2 Table).

Based on the possession of pathotype marker genes, 57 *E*. *coli* strains were classified as EAEC with *aggR* (n = 33, 54.1%), EPEC with *eae* (n = 20, 32.8%), two EIEC with *ipaH* (n = 2, 3.3%), one ETEC with both *elt* and *est*, and one ETEC/EIEC hybrid carrying *elt*, *est* and *ipaH* (n = 1, 1.6% each) (Fig 1). The PCR results for ECV425 was *ipaH*-positive, while the genome analysis was *ipaH*-, *lt*-, and *st*-positive. All other strains indicated no difference between PCR and genomic analysis (data now shown). Among EAEC, ten strains formed the dominant group showing ST31 in phylogroup D and Og130:Hg27, and five (15.2%) formed the second dominant group showing ST3570 in phylogroup B1 and OgX13:H27 (n = 4) and OgL19:H27 (n = 1) (Fig 1). Among EPEC, five strains showed ST40 in the B1 phylogroup and Og51:Hg21 (n = 3), Og115:Hg21, and OgX18:Hg21 (n = 1 each), and four showed ST10 in phylogroup A and OgGp7:Hg16 (OgGp7 is a group consisting of O2 and O50). Two EIEC strains were closely related and classified as OgUT:Hg16. One ETEC and one ETEC/EIEC hybrid were closely related and classified as ST155 and OgUT:Hg21.

### Other virulence-related genes

In addition to major virulence markers, other virulence-related genes were also confirmed in the genomes. Focusing on some representative genes (Fig 1), the *hlyA* encoding α-hemolysin and *invX* related to invasion were carried by two and one strains, respectively. The *astA* gene encoding enteroaggregative stable toxin 1 (EAST1) and *set1A* encoding *Shigella* enterotoxin 1 (ShET1) were confirmed in 32.7% (n = 20) and 45.9% (n = 28) of the strains, respectively. The *cdtB* gene encoding cytolethal distending toxin (Cdt) was identified only in four *E*. *albertii* strains. The *bfp* gene involved in typical EPEC was not confirmed in any strain.

### Antibiotic resistance gene and point mutation

**Fig 2:**
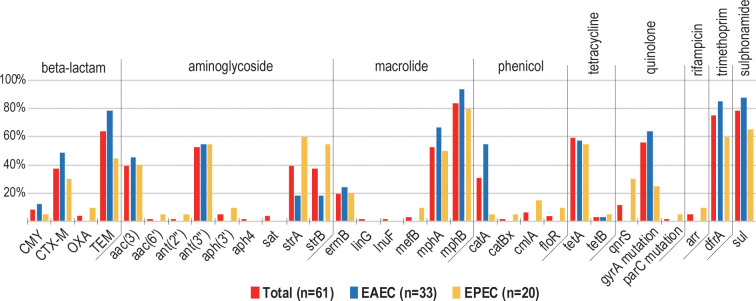
Prevalence of antimicrobial resistance genes/mutations.

All DEC strains except one (EVC1009-1) had some antibiotic resistance genes (Fig 1). One or more beta-lactam resistance genes including *bla*CMY (n = 5), *bla*CTX-M (n = 23), *bla*OXA (n = 2), and *bla*TEM (n = 39) was carried by 78.7% of strains (n = 48) (Figs 1 and 2). The *bla*SHV gene was not detected in any strains. It was found that 41% of strains (n = 25) harbored genes associated with extended-spectrum beta-lactamase (ESBL) production, including CMY-42 (n = 2) and CTX-M, which is further divided into five CTX-M molecular types, namely, CTX-M-14 (n = 2), CTX-M-15 (n = 2), CTX-M-27 (n = 11), CTX-M-55 (n = 7), and CTX-M-65 (n = 1) (S2 Table). Aminoglycoside resistance genes were carried by 68.9% (n = 42) of the strains (Fig 1). The prevalence of some aminoglycoside resistance genes including *strA*, and *strB*, was found to be significantly higher in EPEC than in EAEC (p < 0.05) (Fig 2). One or more macrolide, phenicol, tetracycline, rifampicin, trimethoprim and sulphonamide resistance genes were carried by 93.4% (n = 57), 41% (n = 25), 37.7% (n = 23), 4.9% (n = 3), 75.4% (n = 46) and 78.7% (n = 48) of the strains, respectively (Fig 1). These findings were further substantiated by assembled draft genomes, except for a few genes (S3 Table). The presence of *qnrS* and point mutations on *gyrA* and *parC* associated with quinolone resistance were confirmed in 65.6% (n = 40) of strains (Fig 1).

## Discussion

The study has clarified the prevalence and repertoires of virulence-related and antibiotic-resistance genes, and the phylogenetic features of DEC and *E*. *albertii* strains isolated from infants with diarrhea in Vietnam. Nguyen et al. [6] investigated the prevalence of DEC in Vietnam using 587 fecal samples from children with diarrhea, EAEC and EPEC were detected in 11.6% and 6.6% of the samples, respectively, and EIEC, ETEC and STEC were only in 2%, 2.2% and 0% respectively. Our study also showed that EAEC (6.5%) and EPEC (4.7%) were the main pathotypes, suggesting that EAEC and EPEC are mainly involved in common infant diarrhea in Vietnam. Among the various types of DECs identified, the presence of several epidemic clones was confirmed. EAEC Og130:Hg27 (ST31, n = 10) was the most abundant type, followed by EAEC OgGp9:Hg18 (ST414, n = 4), EAEC OgX13:Hg27 (ST3570, n = 4), EPEC OgGp7:Hg16 (ST10, n = 4), EPEC Og51:Hg21 (ST40, n = 3), and *eae*-positive *E*. *albertii* EAOg1:HgUT (n = 3). These were isolated from the samples of different patients. Among 503 EAEC strains registered in EnteroBase (https://enterobase.warwick.ac.uk/), 9 strains belonging to ST31 (which were isolated in the UK, Germany, Nigeria, Peru, Thailand, and Bangladesh), 2 strains of ST414 (UK), and 1 strain of ST3570 (Germany) were identified. In the 218 EPEC strain information, ST10 was confirmed in 8 strains (Norway, UK, Germany, Brazil) and ST40 in 7 strains (Norway and U.S.A.). Although there have been some reports of EAEC O130:H27 being isolated from sporadic diarrheal cases in Thailand, Peru, and England [37–39], no epidemics due to EAEC O130:H27 have been reported worldwide. Since OX13 is an atypical O-serogroup that does not fall under the defined *E*. *coli* serotypes O1 to O188, a few characteristics of strains belonging to OX13 that can be shared have been accumulated so far. Detailed genomic analysis of strains isolated from infants in Vietnam suggests that some DEC clones, which have not yet become a public health problem worldwide, may be prevalent in Vietnam. *E*. *albertii* is an emerging diarrheagenic pathogen that was first isolated from the feces of diarrhea infants in Bangladesh in 1991 [40]. The basic pathogenicity of *E*. *albertii* is the formation of A/E lesions by locus of enterocyte effacement (LEE), as in EPEC. Initially, *E*. *albertii* was classified as *Hafnia alvei* but was subsequently proposed as a new species, “*E*. *albertii*”, belonging to the genus *Escherichia* in 2003 based on several genetic and phenotypic analyses [41]. Since then, *E*. *albertii* has been implicated in several diarrheal cases including outbreaks [42–45]. The biochemical properties of *E*. *albertii* are very similar to those of *E*. *coli* except for a few properties, such as motility and fermentability of some sugars. Therefore, *E*. *coli* and *E*. *albertii* are sometimes indistinguishable by basic biochemical tests. Furthermore, since *E*. *albertii* usually carry the LEE-carried *eae*, it is presumed that *E*. *albertii* are often misidentified as EPEC. Also, in this study, four strains carrying *eae*, which were thought to be EPEC, were identified as *E*. *albertii* via genomic analysis. They have also acquired resistance to various antimicrobial agents, like DEC strains. These results suggest that *E*. *albertii* is one of the causes of infant diarrhea in Vietnam. This is the first report of *E*. *albertii* isolation from patients in Vietnam. Further information is required to be collected to understand the distribution and epidemiology of this emerging diarrheagenic pathogen.

The spread of ESBL-producing *Enterobacteriaceae* showing resistance against broad-spectrum beta-lactam antibiotics poses a threat worldwide [46]. In Vietnam, many investigations reported the presence of ESBL-producing *E*. *coli* in animals, foods, the environment, and healthy humans [47–52]. Truong et al. [50] investigated the *E*. *coli* strains isolated from workers and pigs at Vietnamese pig farms, and they confirmed that 74% (43/58) and 90% (78/87) of isolates from workers and pigs, respectively were ESBL-producing *E*. *coli*. Nakayama et al. [49] investigated chickens in Vietnam and isolated ESBL-producing *E*. *coli* from 90% (54/60) of samples. Lien et al. [51] revealed that 43% (76/158) of *E*. *coli* strains isolated from hospital wastewater in Vietnam were identified as ESBL-producing. In contrast, there are no reports of ESBL-producing *E*. *coli* isolated from patients, except for a few extraintestinal infections [52]. In this study, we identified that 41% of DEC from patients with diarrhea in Vietnam were potentially ESBL-producing, and especially two *bla*CTX-M types, CTX-M-27 and CTX-M-55 contributing to ESBL production were widely distributed in DEC. Recently, Robert et al. [53] reported the genomic analysis of 721 *E*. *coli* strains isolated from patients and environments in ICUs at two hospitals in Hanoi, Vietnam. It is unknown whether the *E*. *coli* isolated from patients including stool, urine, and sputum etc. are pathogenetic, however, 85, 29, and 48% of them harbored *bla*CTX, *bla*CMY, and *bla*TEM, respectively. The major types were CTX-M-15 (36%), CTX-M-27 (30%), CTX-M-55 (17%), which were correspond to the findings in this study. However, the ICU strains belonged to 80 sequence types including ST410, ST617, ST131, ST648, and ST1193, and no major overlap with the DEC strains used in this study was identified.

The widespread presence of antibiotic-resistance bacteria is a severe and growing public health issue. According to the pediatric diarrhea treatment guideline in Vietnam uploaded in 2016 (https://kcb.vn/), the use of two antibacterial agents, ciprofloxacin belonging to the second-generation fluoroquinolone and ceftriaxone belonging to third-generation cephalosporin, is recommended for the treatment of infantile diarrhea. However, it was estimated that 65.6% (quinolone resistance) and 41% (ESBL-producing) of DEC and *E*. *albertii* in this study were expected to be resistant to these antibiotics. The evolution of ciprofloxacin resistance in *E*. *coli* involves the accumulation of point mutations on *gyrA* and *parC*. By combining experimental data and mathematical modeling, Huseby et al. [54] showed that the first step in the evolution of clinical ciprofloxacin resistance was the selection of *gyrA* mutations and one main trajectory that leads to clinically relevant resistance was S83L. A single S83L mutation had an MIC of about 0.25 mg/ml (10 to 20 times that of the wild-type strain) against ciprofloxacin, and additional point mutations of different sites on these two genes made it more resistant [54]. A large-scale genome analysis focused on *Shigella sonnei* showed that sequential accumulation of mutations in *parC* and *gyrA*, including *gyrA*-S83L, led to the emergence of fluoroquinolone-resistant *S*. *sonnei* in approximately 2007, this was then the population spread worldwide [55]. Ciprofloxacin and ceftriaxone have been used following the standard Vietnamese treatment guidelines for diarrhea patients even before the guideline was updated in 2016 [56], and most of the dominant DEC, including EAEC Og130:Hg27 and EPEC OgGp7:Hg16, and all *E*. *albertii* carried genes conferring resistance to these two antibiotics, suggesting that the routine use of antibiotics according to the guideline has facilitated the selection of resistant strains. The resulting situation is one in which they cannot contribute to improving therapeutic effects for some patients. Bridging this gap requires continuous investigations and information sharing regarding the type and distribution of antibiotic resistance in each pathogen, including DEC and *E*. *albertii*, from each region and country.

## Conclusion

In summary, this study reports the genomic analysis results of DEC and *E*. *albertii* isolated from infant diarrhea patients in Vietnam. We found some DEC clones, including EAEC Og130:Hg27, EAEC OgGp9:Hg18, EAEC OgX13:H27, EPEC OgGp7:Hg16, and *E*. *albertii* EAOg1:HgUT circulating in the area. Most strains possessed several antibiotic resistant genes and mutations, suggesting they have acquired multidrug resistance capacity. Furthermore, many were speculated to be resistant or potentially resistant to ciprofloxacin and ceftriaxone, which are widely recommended drugs for diarrhea treatment in children. The divergence between the distribution of antibiotic resistant strains and the types of antibiotics recommended makes it challenging to improve the effectiveness of antibiotic treatment against infections. We believe that continuous investigations and information sharing of antibiotic resistance in pathogenic bacteria, including DEC, in Vietnam are necessary to improve the therapeutic effects of antibiotics in patients.

## Supporting information

S1 Table Overview of 122 DEC strains isolated in this study and selection the process. (XLSX)

S2 Table List of DEC and *E*. *albertii* used in this study and details of analysis results. (XLSX)

S3 Table Results of homology search for drug resistance genes using assembled genomes. (XLSX)
